# The Influence of Self-Concept on Clinical Decision-Making in Nurses and Nursing Students: A Cross-Sectional Study

**DOI:** 10.3390/ijerph17093059

**Published:** 2020-04-28

**Authors:** Nikolina Farčić, Ivana Barać, Robert Lovrić, Stana Pačarić, Zvjezdana Gvozdanović, Vesna Ilakovac

**Affiliations:** 1 Nursing Institute “*Professor Radivoje Radić*”, Faculty of Dental Medicine and Health Osijek, Josip Juraj Strossmayer University of Osijek, 31 000 Osijek, Croatia; 2 Department of Surgery, University Hospital Centre Osijek, 31 000 Osijek, Croatia; 3 Faculty of Medicine, Josip Juraj Strossmayer University of Osijek, 31 000 Osijek, Croatia; 4 General County Hospital Našice, 31 500 Našice, Croatia

**Keywords:** CDMNS, clinical decision-making, hospital nurses, nursing students, nurses’ self-concept, NSCQ, work experience, education

## Abstract

The aim of this study is to examine the influence of nurses’ self-concept (NSC) on clinical decision-making (CDM) among nursing students and hospital nurses. A further aim is to examine whether there is a difference in CDM and NSC between hospital nurses with various levels of experience and nursing students. A cross-sectional study was conducted during 2018 in the Faculty of Dental Medicine and Health and the University Hospital Center, in Osijek, Croatia, EU. The respondents consisted of 568 hospital nurses, and 129 BSc nursing students. Data were collected with the clinical decision-making in nursing scale (CDMNS) and the nurses’ self-concept questionnaire (NSCQ). There was no correlation between CDM and NSC in either students or hospital nurses. Hospital nurses generally had significantly higher scores in CDM than nursing students. On the other hand, students had a significantly higher total NSC level. The results of this study suggest that experience has a positive impact on nurses’ CDM skills. The high NSC estimated by students enables them to easily take up their clinical roles and approach the patient in a holistic manner, which is an attitude that gradually develops during studies and with clinical experience.

## 1. Introduction

Clinical decision-making (CDM) in nursing practice is a complex process integrated into the nursing profession [1,2,3]. CDM is an essential skill that a nurse uses in clinical practice [4], results from critical thinking [5], involving both knowledge and clinical experience and is defined as the process of choosing alternatives in providing care to patients [6]. The decisions nurses make affect patient care, safety, and outcomes [7,8,9]. Analysis of the relevant literature in the area of nursing indicates a lack of research papers that question the correlation between nurses’ self-concept (NSC) and CDM in the earliest stages of acquiring nursing competencies in tertiary education and in the later phases of applying those competencies in a real clinical environment. This study contributes to a better understanding of CDM in nursing students and hospital nurses working in a clinical environment, and further clarification of the influence of NSC on CDM.

Benner [10] developed an intuitive, humanistic decision-making model, called “From Novice to Expert”, in which she described five stages of skill acquisition in nursing clinical knowledge, namely novice, advanced beginner, competent, proficient, and expert nurse. These five phases reflect changes in three general aspects of the successful performance of acquired skills and CDM: shifting from relying on abstract principles toward using past concrete experiences, changing the perception of a situation from specifying a few specific parts to seeing a more complete picture, and moving from a position of a remote observer to an active performer of nursing skills. Each level is characterized by reliance on past clinical experience [10]. Benner states that nurses with more than five years of work experience are developing an intuitive pattern for CDM [10]. Intuition is defined as a pattern matching, another cognitive strategy that helps build knowledge from previous clinical experiences [10]. Pattern matching, from previous experiences, influences nursing practice [11,12]. However, intuition, used by expert nurses, may lead to errors because of the influence of experience-based preconceptions [11]. In contrast, one study found that using rationale as a basis for decision-making, analytic decision-makers were more experienced, were older, and had worked longer in their unit [13].

According to O’Neill et al., 2005 decision-making is multidimensional, they suggest that the complexity of CDM requires a broad knowledge base, access to reliable information as well as working in a supportive environment [14]. Education, situation awareness, autonomy [3], as well as the freedom to make the right decisions are also important. Nursing and medical knowledge have increased dramatically, and accordingly, decision-making and nursing competencies in healthcare have modified rapidly and continue to do so [15]. Increased patient acuity, reduced length of patient hospital stays, and constant advances in technology have been factors contributing to the need for nurses to make decisions quickly [16]. 

CDM is part of the nursing process, which consists of assessment, identifying the health problem, diagnosis, planning, implementation, and evaluation, so that the clinical decision would be effective [17]. Students learn theoretical knowledge and practical skills so that they can develop competencies for different aspects of nursing work [15]. Competencies of a nurse include knowledge, skills and attitudes, but also critical thinking, so they can successfully manage their work in an ever more demanding clinical practice [18]. In order to achieve autonomy in nurses’ decision-making, development of the intellectual and cognitive skills needed for data analysis and critical thinking [19] must be introduced in their education and continuous training [20]. 

Hoffman et al. [21] discussed the importance of different factors influencing CDM. These factors are experience, level of education, value of the role played by the professional, area of specialty, hierarchy, stress, confidence, and personal beliefs. The conclusion of the mentioned research study was that the value of the role played by the professional is the most influential factor in CDM [21]. Professional values are goals and beliefs about the appropriateness and desirability of something in a profession and provide a basis for decision making [22]. Nursing performance is based on professional ethics and ethical values and core values of nursing include honesty, altruism, human dignity, autonomy and social justice [23]. Values are innate and flexible, and they might change over time [24]. They can be taught, might be modified and promoted through education [25]. The nursing educators and continuous education play a key role in learning and instilling the professional values [26]. One study showed that the professional values’ scores of the nursing students were significantly higher than nurses [27], which indicates that values learned in the education programs might be changed after employment.

The professional NSC can be described as the nurses’ own perception of their duties, knowledge and behaviors that helps them to develop their professional values [28]. Nurses with a strong sense of NSC can achieve changes likely to positively influence themselves, as well as their patients, colleagues and the whole profession [29]. Nurses with a strong sense of NSC can provide high quality nursing care and represent the nursing profession in the community [29]. Nurses’ self-concept is affected by working conditions, by their personal characteristics [30] and it depends on the professional experience in a clinical environment. Losa Iglesias et al. have shown that professional experience leads to increased self-esteem and reduced feelings of stress, anxiety, and burnout [31]. Nurses with higher self-esteem had better performance in unpleasant situations. Also, level of self-esteem and NSC can affect effective communication with patients [32]. Higher and stable levels of NSC may lead to increased retention rates in the healthcare workplace [33,34], high job satisfaction, and low burnout levels [35]. Insufficient NSC can lead to failure to successfully fulfil the nursing role or meet professional standards, job dissatisfaction, burnout and resignations [35]. Education designed with the purpose of developing students’ professional skills, together with providing a positive image of the nursing profession, can increase students’ NSC [36]. 

NSC may vary in critical ways during the working life of nurses. It is believed that the transition from a student to a graduate nurse forms the critical point in a nursing career [37]. This is a period when a novice finds out whether their chosen occupation is congruent with his/her own self-concept. Triumphing over these initial difficulties allows the experienced nurse to have a higher NSC, in fact, early positive experiences can help the nurse when he/she is an experienced nurse [38]. The development of NSC will improve their clinical performance [39]. The education environment affects student learning and development, students perceived that the program increases their self-confidence, and helps them develop their professional skills [40].

Various studies have concluded that nursing students are not prepared for real practical work and current educational programs are insufficient [41]. They feel unprepared with the responsibility of their professional role [42,43]. Nursing students need to develop highly professional NSC, a sense of responsibility for their profession, patients and themselves, and later, be willing to apply, expand and adapt their knowledge to each patient’s needs, quickly and properly. A higher professional self-concept was associated with a reduction in the level of stress among the students, and lower NSC can be one of the possible causes of stress among the nursing students [44]. Confident nurses are initiators, not only followers, and make preventative decisions instead of simply responding to problems [45]. Therefore, it is necessary to determine the perception of nursing students and experienced nurses in CDM with regard to their NSC.

As far as we know, our research is the first one that examines the correlation of NSC and CDM in nursing students and hospital nurses. Adding to the existing literature, the aim of this study is to examine the influence of NSC on CDM among nursing students and hospital nurses, and whether there is a difference in CDM and NSC between hospital nurses with various levels of experience and nursing students.

## 2. Materials and Methods 

### 2.1. Study Design and Participants

A cross-sectional study was conducted from March to August 2018. The research was conducted in the Nursing Institute “Professor Radivoje Radić”, Faculty of Dental Medicine and Health and the University Hospital Center, in Osijek, Croatia, EU. The strengthening the reporting of observational studies in epidemiology (STROBE) checklist for cross-sectional studies was used in reporting this study [46].

The participants were selected using the principle of availability according to defined criteria. There were 697 respondents who participated in the study, who were divided into two groups.

The first group of respondents consisted of 568 registered nurses employed in the hospital, at clinical (not intensive) departments, who are in direct contact with patients and provide 24-h healthcare. The participants were selected using the principle of availability. The criteria for inclusion of nurses in the research were at least one year of work experience, a permanent employment contract, and voluntary consent for participation in the research. Exclusion criteria included longer absences from work (e.g., for sick or maternity leave), change of workplace over the past six months, and nurses who work at departments where they do not have continuous contact with a patient (quality departments, transfusiology, departments of intrahospital infection and sterilization), as well as those who are leaders and are dealing with management rather than health care. Registered nurses with less than a year of work experience (novices and advanced beginners) were also excluded from the research because they had temporary contracts.

The second group of respondents were 129 nursing students of the second and third year of a bachelor’s degree program (because those nurses gained previous experience of real clinical practice with real patients, through obligatory clinical practices), occupational unemployment, and voluntary consent to participate in the research. The participants were selected using the principle of availability.

#### Sample Size

The sample size was calculated using the online software “Sample Size Calculator” from Creative Research Systems [47]. The calculation was based on the total number (920) of nurses at the University Hospital Center Osijek, with initially defined values of confidence interval (margin of error) of 3%, confidence level of 95% and α level of 0.05. The calculation of the least sample size for this study was 494 subjects. A total of 600 questionnaires were distributed to nurses, of which 568 were analyzed because 32 questionnaires were not completely filled out. Furthermore, the sample size of 129 students was determined by the total number of students enrolled in the second and third years of their academic year 2018/2019. Thus, all 129 (100%) enrolled students volunteered for this study.

### 2.2. Study Instrument

For this study, a specific instrument was selected based on the overall appropriateness of the instrument for measuring the intended study variables. Also, specific instrument was selected based on the instrument’s psychometric properties, length of time required to complete the questionnaire, and availability [48]. The instrument consists of two sections, the first one gathers general participants’ information, and for the nurses this included: age, gender, level of education and years of work experience in nursing. For the students, general participants’ information included: age, gender, year of study, and occupational employment. The second part of the study instrument consists of two validated questionnaires: The clinical decision-making in nursing scale (CDMNS), and the nurses’ self-concept questionnaire (NSCQ). For the purpose of selection, translation, validation and cross-cultural adaptation of the instruments, the following procedures were applied: selection of instruments, obtaining permission from the authors, translation and back-translation, language proofreading, evaluation of the statistical reliability and content validity of the questionnaires [49,50].

#### 2.2.1. Clinical Decision-Making in Nursing Scale (CDMNS)

The CDMNS [51] is a validated questionnaire for examining nursing decision-making, measures the choice of information strategies for decisions. The questionnaire consists of a total of 40 questions that represent four subscales: (1) search for alternatives or options, (2) canvassing of objectives and values, (3) evaluation and re-evaluation of consequences, and (4) search for information. Each of the sub-scales contains ten questions that are assessed according to 5-point Likert scale, ranging from 1 (“never”) to 5 (“always”), which gives a potential score range of 40–200 in the whole scale and 10–50 in the subscales, and there is no cutting point. Higher scores indicate a positive perception of decision-making, and lower scores, on the contrary, indicate a negative perception of decision-making. Additionally, in the survey presented by Canova et al. 2016 the total scoring of CDMNS is divided into low (40–130), medium (131–160), and high (161–200) perception of CDM [52]. The internal consistency reliability of the CDMNS was established with a sample of nursing students and yielded a Cronbach’s alpha coefficient of 0.83 [38], and as a result, it has been used in over ninety research studies so far [52,53]. Regarding the sample of participants in this study, the reliability of the internal consistency (Cronbach’s alpha) is high and amounts to 0.85 for the whole scale.

#### 2.2.2. Nurses Self-Concept Questionnaire (NSCQ)

The NSCQ [54] is a questionnaire designed specifically for the examination of nurses’ self-understanding with regard to their professional role. The questionnaire contains a total of 36 statements that represent six categories: (1) general self-concept, (2) nursing care, (3) staff relations, (4) communication, (5) knowledge, and (6) leadership. Each sub-scale has six statements that the respondents’ rate on an 8-point Likert scale, ranging from 1 (“completely incorrect”) to 8 (“totally accurate”), making a potential score range of 36–288. All claims are positively formulated, and each subscale contains the balance of affective and cognitive declarative claims. Higher scores are interpreted as the highly developed self-concept of a nurse. Hensel et al. 2010 conducted a study using this instrument and reported a Cronbach’s alpha of 0.87–0.91 for its various dimensions [55]. Regarding the sample of participants in this study, the reliability of the internal consistency (Cronbach’s alpha) is high and amounts to 0.91 for the whole scale.

### 2.3. Data Collection Procedures

The sample was composed of hospital nurses and nursing students, recruited in two ways. The researchers distributed a questionnaire in the clinical departments to all nurses. Nurses were invited to participate in this study by voluntarily completing and returning the questionnaire in sealed envelopes. Surveying among nursing students was conducted in a prearranged time, before or after the lectures or seminar classes.

### 2.4. Ethical Considerations

The ethical committee of Faculty of Dental Medicine and Health and Osijek University Hospital (Approval number: R1-20205-2/2017) approved this study. All subjects were informed about its aim, and they gave informed consent to participate in the research on a voluntary basis. The anonymity of the participants was guaranteed. The study was conducted in accordance with the Declaration of Helsinki.

### 2.5. Data Analysis

Numerical data are expressed as mean values and standard deviation. Parametric tests, ANOVA (between four nursing groups) and Student’s *t*-test (between nurse and student groups) were used to make a general comparison of all score distributions. The correlation analysis evaluated, using the Pearson’s correlation coefficient, the relations between the NSCQ and the areas of the CDMNS. The reliability of the questionnaire was verified by the Cronbach’s alpha coefficient. All statistical analyses were performed using the Statistical Package for Social Sciences (SPSS) version 15.0 (SPSS Inc., Chicago, IL, USA). In all the analyses, a *p*-value less than 0.05 was considered statistically significant.

## 3. Results

### 3.1. Sociodemographic Characteristics

There were 697 (100%) respondents who participated in the study, out of which 568 (81.5%) were full-time employed hospital nurses, and 129 (18.5%) were nursing students (Table 1). There were no participants with missing data. The mean value of the hospital nurses’ work service was 19.84 (SD 11.7) years, divided into four categories: ≤10 years (145 nurses), 11–20 years (156 nurses), 21–30 years (141 nurses), and >31 years (126 nurses). Student respondents did not have occupational work experience.

### 3.2. CDM and NSC by Years of Nursing Work Service

Nurses with more than 31 years of work experience had a significantly higher score (*p* < 0.001) in decision-making (mean = 129.87; SD 17.84), as well as higher scores in all subscales (*p* < 0.05), compared to nurses with less work experience. Hospital nurses compared to nursing students generally had a higher score in decision-making (*p* = 0.003), as well as in all subscales (*p* < 0.05). The total score of the assessments made by all the categories of the respondents were in the lowest class (0–130) of the CDMNS (Table 2).

Regarding NSC results in nurses and students, the group of nurses with 11–20 years’ experience had the lowest NSC level compared to the other three groups of nurses, in three subscales: general (self-concept) SC (*p* = 0.039), knowledge (*p* = 0.032), and leadership (*p* = 0.037). The group of nurses with >31 years’ experience had the highest NSC level (mean = 230.7; SD = 31.7) compared to the other groups of nurses, but without statistical significance.

Nursing students had a significantly (*p* < 0.001) higher level of total NSC (mean = 247.56; SD 21.79) compared to nurses and gave significantly higher scores (*p* < 0.001) in five subscales: General SC, staff relations, communications, knowledge and leadership (Table 3). In the nursing care subscale, there was no difference in either category, whether the respondents were hospital nurses or students.

### 3.3. The Influence of NSC on CDM among Nursing Students and Hospital Nurses

There was no statistical correlation between variables of total NSC and overall CDM (r = 0.178 **, *p* < 0.01) in nurses (Table 4). There was a weak positive correlation between the CDMNS canvassing subscale and total NSC (r = 0.275 **; *p* < 0.01) and the general SC (r = 0.251 **; *p* < 0.01). The nursing student NSCQ data showed a statically insignificant correlation between CDMS data (Table 5).

## 4. Discussion

Novice BSc nurses must be able to effectively make complex decisions about patient care upon entering practice [56]. In this study, a weak positive correlation was found between the total and general NSC and the CDM “canvassing of objectives and values” subscale that focuses on the professional values and attitudes of decision-makers in terms of diversity. This also refers to the holistic approach to a patient. These research results are in accordance with Benner’s theory, confirming that only expert nurses are able to step back and see the patient as a whole, rather than as a series of tasks that need to be performed in nursing care. This, according to Benner, is part of the progress in decision-making [10]. Pattern matching from previous experiences influences nursing practice [11,12]. However, intuition used by expert nurses may lead to errors because of the influence of experience-based preconceptions [12]. The results of this study also indicate that there is no correlation between the variables of total NSC and overall CDM in hospital nurses and nursing students. This discrepancy in results could be apparent because of the “gap” between theory and concrete clinical practice, as well as between curricular training and autonomous work in everyday real situations [57].

The results of this research indicate that nursing students and hospital nurses have the ability to make decisions in a clinical environment but assess this ability as low [51,52] (in the 40–130 score range). In this study, looking for alternative solutions is one of the major attributes when making clinical decisions. The results of this study are in agreement with the results of the study conducted by Ho et al. [58]. Research conducted among Italian nurses and nursing students from 1997 to 2012 showed that total clinical decision-making scores were lower each year since 1997, in both categories of respondents, ranging from 118 (SD 14) in 1997 to 94 (SD 13) in 2012. Nurses, in comparison with students, had higher CDM scores, and experienced nurses had higher CDM scores than ones with less professional work experience [52].

With regard to clinical experience, the results of this study indicate that nurses with more work experience held a more positive perception of their CDM. Thus, participant nurses with more than 31 years of work experience had the highest CDM scores, compared to less experienced nurses and students. The results of this research also indicate that the CDM scores assessed by nurses at the beginning of their professional career is lower than the value assessed by students (mean = 123, SD 13.7), but it grows over the years as the nurses become more experienced. The Italian research mentioned above is interesting because it showed that, in 1997, nursing students had greater CDM values than nurses, although this result was not confirmed in their next two surveys (2007 and 2012). The authors explained such results as the outcome of changes in the Italian system of tertiary nursing education [52]. This “gap” between education and clinical practice is considered to be an immediate “consequence” of the way that clinical training and study programs are organized in nursing educational institutions [59]. After completing their education, nurses come to clinical departments full of self-confidence in their competences, but as they begin to work, they realize that they are “thrown” into responsible professional roles they cannot cope with [60].

In a 2016 study by Wu et al., experience had a significant positive impact on nurses’ CDM skills [61]. However, in several other studies, results indicated that experience is not related to decision-making [48,62].

In this study, nursing students assessed a significantly higher value of NSC in comparison to full-time employed nurses. This result is in clear contradiction to the findings of Cowin et al. who suggested that experienced nurses have higher NSC than nursing students and new graduates [38]. The authors also concluded that NSC fluctuates at the development stage from students to graduate registered nurses but remains stable for experienced nurses [38], which is in accordance with results of this research.

The results of this study also show that the value of the score on the NSCQ at the beginning of nurses’ professional career is lower than the students’ scores, but then it grows over the years of work experience. During their studies, nursing students respect their profession and have a high opinion of themselves as professionals, they absorb a lot of theoretical knowledge and are confident about how they will apply it. Starting to work in a real clinical environment, they are faced with many challenges, such as hard-working conditions, individual patient needs, new technology, communication requirements, quick decision-making, and unpredictable situations, and the level of their NSC decreased. So, it is important to develop the concept of self as a professional nurse already during occupational education [63]. Nevertheless, the results reveal high evaluations of the NSC in nursing students, which later on, during a professional career, can contribute to their constant desire to learn and to improve their professional skills, and thus prevent stress-related burnout at work [64]. Moreover, it is possible that excessive self-confidence leads to the use of poor information strategies such as gathering, sharing, communicating, and understanding of this information, and to decisions without full data collection and analysis [65]. However, too much confidence in one’s abilities are not always welcome, because they may lead to quicker decisions which are not necessarily more accurate [66].

The results of this study also indicate that although the values of all subscales of the NSCQ decrease after full-time employment, the nursing care subscale remains stable in all categories of nurses and students.

It is necessary for clinical nurses to be ready for problem-solving and gaining new experiences in decision-making. This cannot be achieved without the initial high-level of NSC, which somewhat lowers (in comparison with one assessed by students) at the beginnings of full-time employment in a clinical environment, but it persists and even rises later on, so nurses can act positively in health care [55]. Effective decision-making is the basic condition for the future of professional nursing practice. Therefore, nursing education and training rest on the responsibility to increase the ability of nurses to make proper clinical decisions [67]. The literature presents the need to strengthen resilience, as well as developing confidence in one’s skills, guidance for career progression, and commitment of both the University and health organization to support staff to be healthy and feel valued, within undergraduate education in the transition to newly qualified nurse [68]. Clinical decision-making is very complex. Thus, it is evident that only nursing knowledge combined with high professional NSC and motivation, developed through clinical experience in a supportive working environment, can enable nurses to make the right clinical decisions [14]. Higher education institutions should seriously consider all the options of minimizing the “gap” between theory and practice, as well as between clinical training and real work in demanding clinical situations. In doing so, the needed balance would be achieved, and the highest quality of education and ultimately nursing care of the patients would be ensured.

### 4.1. Implications for Nursing Education and Practice

The results of this study provide new insights that can enable the management of hospitals and institutions offering tertiary nursing education to obtain a better understanding of decision-making mechanisms in nurses and students and their self-perception as professionals. In addition, the results could help to identify the factors that cause fluctuations in their developing ability to make the proper decision and to define priorities, procedures, and possible changes in the organization of the departmental work and clinical training. Concrete changes would include: (1) organizing clinical training and mentoring of students according to the “one student, one mentor” model, (2) providing continual nursing education programs on the latest findings and improved practices in health care, (3) implementing existing educational programs with advanced clinical activities, and (4) encouraging the development of highly professional NSC through work independence empowered by critical thinking, problem-solving, and decision-making abilities. The supportive work environment will help nurses and students to develop higher levels of motivation, a more positive perception of their CDM ability and general satisfaction with work in clinical settings. Ultimately, it would have a significant positive impact on nurses and students’ relations with staff and patients and their will to acquire competencies and to learn continuously.

### 4.2. Limitations

By conducting a region-oriented, cross-sectional study, we acknowledge its certain limitations. The study was conducted only in one hospital and one university which may limit generalization of these findings to the entire Croatian nursing population and it is also similar to other studies [21,32,58]. In addition, participants’ responses may be biased regarding with various needs for their decision-making skills because they are employed in different hospital departments, possibly doing their job in different working conditions. The data collected were based on the nurses’ own statement, which we consider as another limitation, what other studies also report [30]. Longitudinal study through nursing school and practice would better describe and explain the development of decision-making abilities over the time. There is a need for more studies exploring the gap between theory and practice, in different contexts and cultures.

## 5. Conclusions

The results of this research suggest that there is no correlation between CDM and professional NSC. Nurses with more than 31 years of work service have a significantly higher decision-making score than nurses with less work experience and students, which suggests that professional experience has a positive impact on nurses’ clinical decision-making skills. On the other hand, students estimate a significantly higher level of total nurses’ self-concept. A high NSC enables students to easily take up their clinical roles and approach the patient in a holistic manner, which is an attitude that gradually develops during study and clinical experience. The results of this study possibly reflect changes in the Croatian system of nursing education, where, despite the major share of the practical training, the student learning outcomes are still mainly focused on the development of factual knowledge and cognitive aspects of their profession. There seems to be a “vicious circle” between theory and practice, and it is necessary to balance the development of cognitive skills with abilities to perform tasks in a real clinical environment.

## Figures and Tables

**Table 1:** Sociodemographic characteristics of the respondents (*n* = 697).

	Sample	Nurses	Students
	*n* (%)	586 (81.5)	129 (18.5)
Gender			
	female (*n* (%))	481 (84.7)	115 (89.1)
	male (*n* (%))	87 (15.3)	14 (10.9)
Age (year)			
	mean (SD)	40.5 (11.7)	26.3 (6.8)
	min–max	20–65	18–44

**Table 2:** Differences in nurses’ decision-making according to their work experience, and between nurses and students.

Nurses’ Experience (Years)	Nurses (*n* = 568)					Differences between Nursing Groups *p* *	Students (*n* = 129)	Differences between Nurses Total and Students *p* **
	≤10 (*n* = 145)	11–20 (*n* = 156)	21–30 (*n* = 142)	>31 (*n* = 126)	Total Nurses (*n* = 568)			
CDMNS	Mean (SD)						Mean (SD)	
Alternatives	32.6 (4.0)	32.4 (4.3)	33.4 (4.3)	34.2 (4.7)	33.1 (4.4)	0.002	33.4 (3.8)	0.320
Canvassing	32.1 (4.2)	31.7 (4.7)	32.2 (4.1)	33.3 (5.4)	32.3 (4.7)	0.03	33.3 (3.9)	0.278
Evaluation	28.1 (4.6)	28.4 (4.5)	28.9 (4.4)	30.9 (5.7)	29.1 (4.9)	<0.001	28.7 (3.7)	0.059
Information	30.0 (4.0)	29.8 (4.0)	29.5 (4.1)	31.3 (5.3)	30.1 (4.4)	0.003	29.9 (3.3)	0.017
Total score	123.0 (13.7)	122.4 (14.8)	124.1 (13.3)	129.8 (17.8)	124.7 (15.2)	<0.001	125.5 (10.0)	<0.003

Legend: CDMNS: Clinical Decision-Making in Nursing Scale; * ANOVA; ** Student’s *t*-test.

**Table 3:** Differences in nurses’ self-concept according to their work experience, and between nurses and students.

Nurses’ Experience (Years)	Nurses (*n* = 568)					Differences between Nursing Groups *p* *	Students (*n* = 129)	Differences between Nurses Total and Students *p* **
	≤10 (*n* = 145)	11–20 (*n* = 156)	21–30 (*n* = 142)	>31 (*n* = 126)	Total Nurses (*n* = 568)			
NSCQ	Mean (SD)						Mean (SD)	
General SC	40.0 (6.9)	37.8 (9.0)	38.7 (7.2)	39.9 (6.8)	39.1 (7.6)	0.039	44.2 (5.5)	<0.001
Staff Relations	38.3 (6.6)	38.1 (6.2)	38.9 (5.9)	39.8 (5.1)	38.7 (6.0)	0.088	41.8 (3.9)	<0.001
Communications	39.1 (6.1)	38.1 (6.3)	39.6 (5.4)	39.5 (5.3)	39.0 (5.8)	0.086	41.9 (3.9)	<0.001
Knowledge	38.8 (6.4)	36.4 (7.2)	38.2 (5.9)	37.9 (6.1)	37.7 (6.5)	0.032	43.2 (3.7)	<0.001
Leadership	31.8 (8.1)	31.2 (9.0)	33.6 (9.3)	33.5 (7.9)	32.5 (8.7)	0.037	36.3 (5.5)	<0.001
Care	40.0 (5.8)	39.2 (6.1)	40.4 (4.9)	40.0 (5.5)	40.1 (5.6)	0.321	40.2 (4.9)	0.418
Total score	227.7 (32.9)	220,9 (37.7)	229.5 (33.3)	230.7 (31.7)	227.0 (34.3)	0.064	247.5 (21.8)	<0.001

Legend: NSCQ: Nurses’ Self-Concept Questionnaire; SC: Self-Concept; * ANOVA; ** Student’s *t*-test.

**Table 4:** Correlation between nurses’ self-concept (NSC) and clinical decision-making (CDM) in nurses (*n* = 568).

CDMNS / NSCQ	NSCQ in Total	NSCQ General	NSCQ Care	NSCQ Communications	NSCQ Knowledge	NSCQ Leadership	NSCQ Staff Relations
CDMNS Alternatives	0.212 **	0.199 **	0.168 **	0.191 **	0.182 **	0.145 **	0.213 **
CDMNS Canvassing	0.275 **	0.251 **	0.199 **	0.248 **	0.236 **	0.226 **	0.243 **
CDMNS Evaluation	0.024	−0.001	−0.010	0.012	−0.007	0.090 *	0.015
CDMNS Information	0.084 *	0.068	0.068	0.065	0.064	0.074	0.095
Total CDMNS score	0.178 **	0.154 **	0.126 **	0.154 **	0.142 **	0.162 **	0.169 **

Legend: CDMNS: Clinical Decision-Making in Nursing Scale; NSCQ: Nurses’ Self-Concept Questionnaire; *p* < 0.05 *; *p* < 0.01 **.

**Table 5:** Correlation between nurses’ self-concept (NSC) and clinical decision-making (CDM) in students (*n* = 129).

CDMNS / NSCQ	NSCQ in Total	NSCQ General	NSCQ Care	NSCQ Communications	NSCQ Knowledge	NSCQ Leadership	NSCQ Staff Relations
CDMNS Alternatives	0.178 *	0.120	0.129	0.134	0.171	0.092	0.208 *
CDMNS Canvassing	−0.023	−0.082	0.000	−0.083	0.077	−0.020	0.021
CDMNS Evaluation	0.094	−0.040	0.043	0.104	0.006	0.219 *	0.093
CDMNS Information	0.139	0.012	0.199 *	0.103	0.124	0.146	0.104
Total CDMNS score	0.139	0.002	0.131	0.090	0.139	0.156	0.156

Legend: CDMNS: Clinical Decision-Making in Nursing Scale; NSCQ: Nurses’ Self-Concept Questionnaire; *p* < 0.05 *.
